# Photovoltage Reversal in Organic Optoelectronic Devices with Insulator-Semiconductor Interfaces

**DOI:** 10.3390/ma11091530

**Published:** 2018-08-25

**Authors:** Laigui Hu, Wei Jin, Rui Feng, Muhammad Zaheer, Qingmiao Nie, Guoping Chen, Zhi-Jun Qiu, Chunxiao Cong, Ran Liu

**Affiliations:** 1 School of Information Science and Technology, Fudan University, Shanghai 200433, China; 17210720055@fudan.edu.cn (W.J.); 16210720056@fudan.edu.cn (R.F.); 17110720056@fudan.edu.cn (M.Z.); gpchenapple@fudan.edu.cn (G.C.); zjqiu@fudan.edu.cn (Z.-J.Q.); cxcong@fudan.edu.cn (C.C.); rliu@fudan.edu.cn (R.L.); 2 Department of Applied Physics, Zhejiang University of Technology, Hangzhou 310023, China

**Keywords:** organic semiconductor, organic photodiodes, organic optoelectronic devices, space-charge, insulator layer

## Abstract

Photoinduced space-charges in organic optoelectronic devices, which are usually caused by poor mobility and charge injection imbalance, always limit the device performance. Here we demonstrate that photoinduced space-charge layers, accumulated at organic semiconductor-insulator interfaces, can also play a role for photocurrent generation. Photocurrent transients from organic devices, with insulator-semiconductor interfaces, were systematically studied by using the double-layer model with an equivalent circuit. Results indicated that the electric fields in photoinduced space-charge layers can be utilized for charge generation and can even induce a photovoltage reversal. Such an operational process of light harvesting would be promising for photoelectric conversion in organic devices.

## 1. Introduction

Organic optoelectronic devices [1,2,3,4,5], including photovoltaic devices [6,7,8,9], usually suffer from space-charges inside them due to poor charge mobility [10] and charge injection imbalance [11], in spite of the advantages of low cost, flexibility and ease of fabrication for organic semiconductors. The space-charges can usually induce a DC photocurrent accompanied by two photocurrent transients with opposite polarities for thick organic thin-film devices [12]. Hu et al. has proposed a double-layer model to explain the space-charge effect [10]. To elaborate, metal/insulator/organic semiconductor/metal (MISM) devices were developed, which were found to be promising for transient photodetection [13], artificial retinas [12,14] and near electric-field detection for ferroelectric domains [15]. The structure can also be utilized for a charge transport characterization in organic semiconductors, i.e., carrier extraction by linearly increasing voltage in MIS structures (MIS-CELIV) [16,17,18,19]. However, the underlying physics of how the space-charges influence device performance is still not very clear, especially for organic photovoltaic devices of which performance can be dramatically enhanced under certain conditions, by the addition of dielectric/ferroelectric components [20,21,22,23,24,25].

In the present work, the physics of how the space-charges in dielectric/organic semiconductor systems change built-in electric fields, electric output and charge distribution was investigated. The photocurrent transients from an MISM device were studied, theoretically and experimentally. An equivalent circuit model was proposed for the MISM devices, which forms a typical sample that has extremely imbalanced charge injection. The “ON” and “OFF” photocurrent transients generated by switching light on and off of a light source were analyzed on the basis of the equivalent circuit. It was revealed that both photocurrent transients show double-exponential decay processes, which can be well explained by the circuit. The collected photo-generated charges in external circuit were found to be only a part of total photo-generated charges. The stored photo-generated charges accumulated at the dielectric/semiconductor interfaces can significantly change the built-in electric fields. It can even screen the fields of a semiconductor, under strong illumination, which induces a zero net field in the bulk region of the semiconductor. Such stored photo-generated charges can even induce a larger field/voltage exceeding the built-in field/potential in a semiconductor layer with an opposite polarity. This can be ascribed to the fields pumping charge continuously within the charge layers accumulated at the interface, due to a large screening length. These give a strong hint that dielectric/semiconductor interfaces with accumulated charges may play a significant role for photoelectric conversion in organic semiconductor devices with dielectric components.

## 2. Materials and Methods

**Figure 1:**
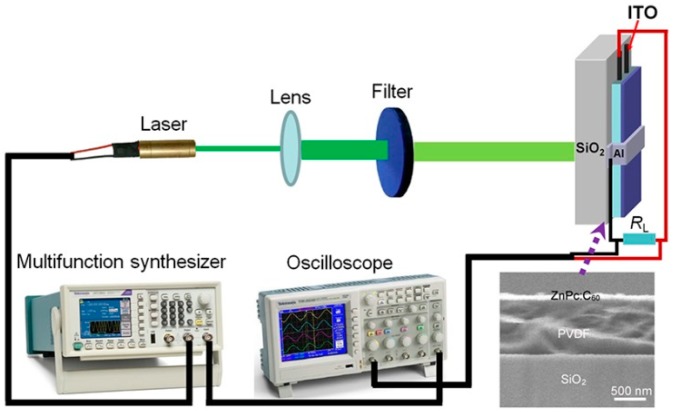
Experimental setup for photocurrent measurements. The inset is a cross-sectional SEM image for a PVDF film covered with a blend film.

Zinc phthalocyanine (ZnPc, 97%) and fullerene (C_60_, 99.9%) were purchased from Sigma-Aldrich (Saint Louis, MO, USA). Polyvinylidene fluoride (PVDF) was obtained from Kunshan Haisi electrical company (Kunshan, China). Glass slides with pre-patterned indium tin oxide (ITO) electrodes were used as substrates, which were treated in an ultrasonic bath with propanol, acetone and chloroform. ZnPc powders were purified by using train-sublimation, and other materials or chemicals were used as received. PVDF films were used as insulator layers (ILs) (around 1 µm); these only exhibit dielectric properties and not ferroelectric ones. The ILs were fabricated by using spin–coating from its solutions (8 wt % in dimethyl formamide) onto hot substrates (100 °C). The thickness of PVDF films were characterized by using cross-sectional scanning electron microscopy (SEM, JEOL Ltd., Akishima, Japan) as shown in the inset of Figure 1. Following this, ZnPc:C_60_ (molar ratio: 1:1) blend films semiconductor layers (SLs) (30 nm) were thermally evaporated onto the ILs in a vacuum of 5 × 10^−4^ Pa at a rate of about 1 Å/s. Film thicknesses were monitored during sublimation using a quartz-crystal microbalance. Finally, aluminum (Al) was also deposited as the cathode by thermal evaporation. The effective area *A* of the MISM (i.e., ITO/PVDF/ZnPc:C_60_/Al) devices was estimated to be 0.02 cm^2^.

Figure 1 shows the experimental setup for our photocurrent measurements. Freshly-prepared samples were fixed in a vacuum chamber with a quartz window for light incidence. The chamber was evacuated into a vacuum with a pressure of <1 Pa. A diode-pumped solid-state (DPSS) laser module (532 nm) controlled by a multifunction synthesizer (300 Hz, Tektronix, Inc., Beaverton, OR, USA) was used as a light source. The laser beam was adjusted by a quartz lens to cover all the effective area of the devices, which were illuminated from the ITO side. Different intensities were obtained by using filters. Before measurements, weak intensity was adopted for laser alignment. Photocurrent transients were calculated based on the photovoltages drop across an input resistance *R_L_* of 10^5^ Ω collected by a high speed oscilloscope (TDS 2002B, 60 MHz, Tektronix, Beaverton, OR, USA).

## 3. Results and Discussions

### 3.1. The Equivalent Circuit of MISM Devices

**Figure 2:**
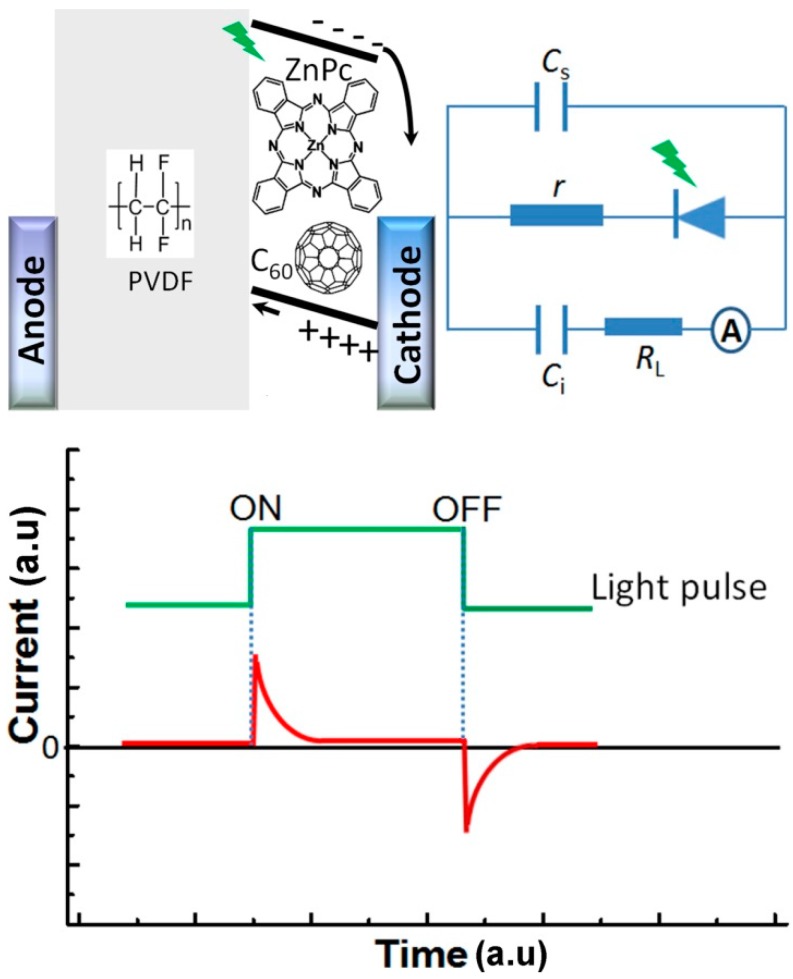
Photocurrent transients from MISM devices. The insets are the schematic views of the double-layer model and related molecular formulae, as well as the equivalent circuit.

To fully understand the MISM devices with photocurrent transients (see Figure 2), an equivalent circuit can be established as the inset. Upon illumination, photo-generated excitons will be formed in the ZnPc:C_60_ film (see Supplementary Figure S1 for details). Assuming that the SL is adjacent to the cathode Al, the photo-generated excitons will be separated into free charges. These can be further extracted by the electrode, due to the built-in electric field caused by work function difference of electrodes under short circuit condition. If the insulator layer is ideal, the DC photocurrent will be prohibited and the holes will accumulate at the IL/SL interface. Photo-generated electrons will be extracted and the charging current for the MISM capacitor can be detected in external circuit with the lights switched on, producing a positive “ON” transient (see Figure 2). Under equilibrium state with illumination, no photocurrent can be collected and charge generation rate would equal to recombination rate in the SL. Part of the photo-generated electrons (*Q_i_*) will be stored in the anode through the charging process. However, the other part (*Q_s_*) will still be left in the cathode to keep a zero electrode potential difference under the short circuit condition, as the SL can be regarded as a photovoltaic source or photodiode with a parasitic parallel capacitance. Therefore, the photodiode can charge the two capacitors under illumination, i.e., the SL and IL capacitors with their capacitance *C_s_* and *C_i_*, respectively. Both the capacitors share the IL/SL interface as an electrode in which the holes are stored. The equivalent circuit can thus be drawn as the inset, which is quite different from the commonly accepted model for MIS diodes under dark conditions, i.e., the IL and SL capacitors are connected in parallel, instead of series, as determined by the device architecture. This is natural, since the parasitic parallel capacitance *C_s_* can be charged by the photodiode even under open circuit condition. This is different from the *C_i_* connected with the diode in series, which can be charged by the diode and also be charged by the *C_s_* under short circuit condition. Therefore, the photodiode is connected with *C_s_* in parallel and *C_i_* in series and this leads on to *C_s_* and *C_i_* being connected in parallel, as was shown in the inset of Figure 2. This means that the current collected in the external circuit is the charging/discharging current of the IL capacitors and not that of the whole MISM devices. Taking into account that the photoinduced charging/discharging current of both the capacitors has to travel through the SL, the internal resistance (*r*) of the SL might also influence the transients. In addition, *C_s_* has an equivalent average value due to the presence of photocurrent as a leakage current. Only under equilibrium states, *C_s_* = *ε*_0_*ε_s_A*/*d_s_*, where *ε*_0_ and *ε_s_* are the vacuum permittivity and the relative permittivity of the SL, respectively; and *d_s_* was the thickness of the SL.

### 3.2. Analyses of the “ON” Transients from the MISM Devices

**Figure 3:**
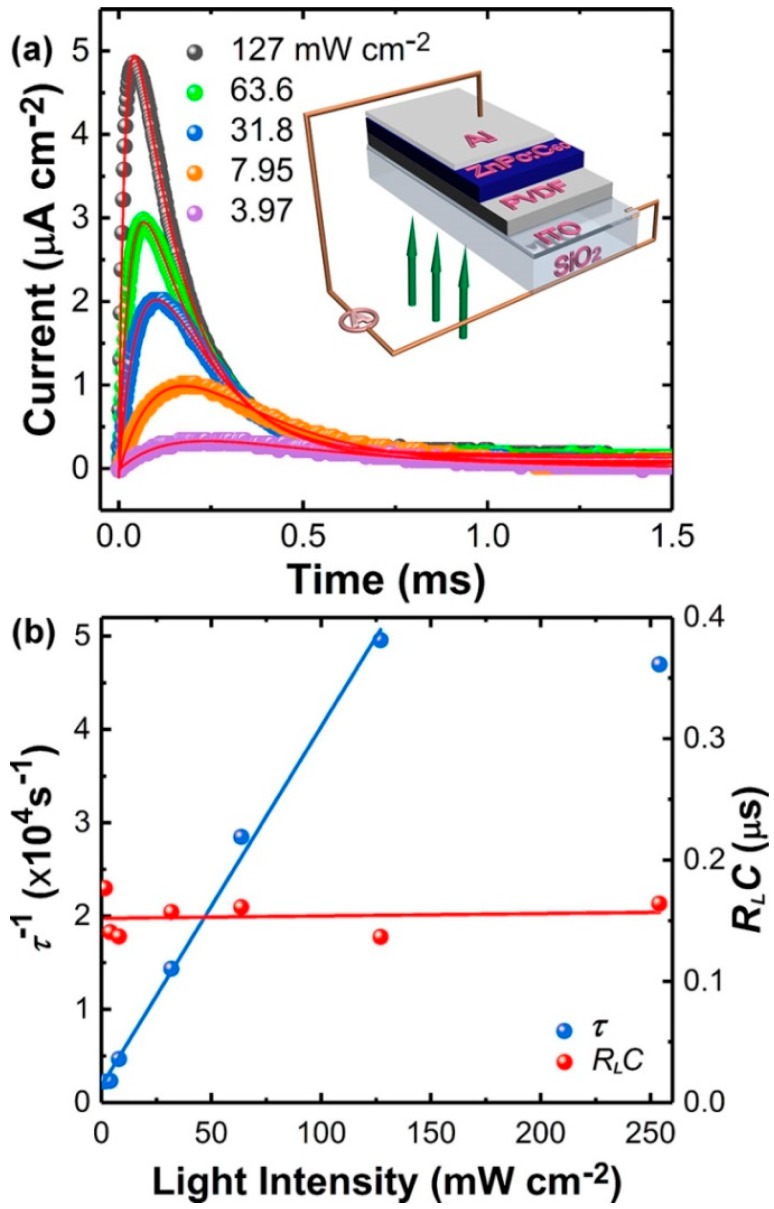
Analyses for the “ON” photocurrent transients from a ITO/PVDF/ZnPc:C_60_/Al device. (**a**) The “ON” photocurrent transients under an illumination from a 532-nm laser with different intensity. The inset is a schematic display for the device. (**b**) Decay time *R_L_C* (red points) and *τ* (blue points) at different light intensity.

Figure 3a shows the “ON” photocurrent transients from an MISM device with different light intensity. All the photocurrent transients *i*(*t*) fitted well with the simulated curves (red) based on the Equation (1):

(1) $$i\left(t\right)=\frac{A𝜉}{\left(𝜏-R_{L}C\right)}\left(e^{-\frac{t}{𝜏}}-e^{-\frac{t}{R_{L}C}}\right)$$

where, *ξ* = *ε*_0_*ε_i_*^2^*d_s_V*/*d_i_* (*d_i_ε_s_* + *d_s_ε_i_*), *R_L_C* is the time constant of the whole circuit and *t* means time. *ε_i_* and *d_i_* are the relative permittivity and thickness of the IL, respectively. *V* is the voltage drop across the whole devices, which can be estimated to be about 0.4 V, and to have been caused by the electrode work function difference. *τ* =*ε*_0_ (*d_i_ε_s_* + *d_s_ε_i_*)/*σ_s_d_i_* and *σ_s_* means the photoconductivity of the SL. Since *σ_s_* = 1/*ρ_s_* = *rA*/*d_s_*, *τ* can be expressed as *r* (*C_s_* + *C_i_*) with *C_s_*_,*i*_ = *ε*_0_*ε_s_*_,*i*_*A*/*d_s_*_,*i*_, where *ρ_s_* is the resistivity of the SL. This indicates that the first term is related to the charging process for both the IL and SL capacitors in parallel, consistent with the proposed circuit. It is notable that one of the two decay times showed light intensity (*I*) dependence (see the blue curve in Figure 3b) and was inversely proportional to *I* if *I* < 127 mW cm^−2^, which can be ascribed to *τ* since *σ_s_* is proportional to *I*, namely, *τ* ∝ *I*^−1^. The other decay time that was independent of *I* could be assigned to the *R_L_C* constant with a value of ~1.6 × 10^−4^ s (see the red curve).

### 3.3. Analyses of the “OFF” Transients from the MISM Devices

The “OFF” current transient, as the discharging current of the IL capacitor, was also studied based on the equivalent circuit in Figure 2. In this case, the photodiode symbol was not considered. Based on the equivalent circuit, the purely photoinduced voltage drop *V_s_*(*t*) across the SL equaled to that across the IL capacitor connected with *R_L_* during the discharging process. Therefore, the time dependent *V_s_*(*t*) would be:

(2) $$V_{s}\left(t\right)=\frac{Q_{s}\left(t\right)}{C_{s}}=\frac{Q_{i}\left(t\right)}{C_{i}}+\frac{dQ_{i}\left(t\right)}{dt}R_{L}$$

Considering that the discharging currents of the SL and IL capacitors were *dQ_s_*(*t*)/*dt* and *dQi*(*t*)/*dt*, respectively, the current *I*_ph_(*t*) through the resistor *r* would be:

(3) $$\frac{dQ_{s}\left(t\right)}{dt}+\frac{dQ_{i}\left(t\right)}{dt}=I_{ph}\left(t\right)$$

Since

(4) $$I_{ph}\left(t\right)r=\frac{Q_{s}\left(t\right)}{C_{s}}$$

two differential equations can be derived as:

(5) $$\frac{dQ_{i}\left(t\right)}{dt}+\frac{Q_{i}\left(t\right)}{R_{L}C_{i}}=\frac{Q_{s}\left(t\right)}{R_{L}C_{s}}$$

and

(6) $$\frac{dQ_{s}\left(t\right)}{dt}+\left(\frac{1}{R_{L}C_{s}}-\frac{1}{rC_{s}}\right)Q_{s}\left(t\right)=\frac{Q_{i}\left(t\right)}{R_{L}C_{i}}$$

By resolving Equations (5) and (6), the discharging current *i*(*t*) collected in the external circuit can be obtained as:

(7) $$i\left(t\right)=\frac{dQ_{i}}{dt}=c_{1}e^{-\frac{t}{{𝜏}_{1}}}+c_{2}e^{-\frac{t}{{𝜏}_{2}}}$$

in which:

(8) $${𝜏}_{1}=\frac{2}{a-\sqrt{a^{2}-4b}},\;\;{𝜏}_{2}=\frac{2}{a+\sqrt{a^{2}-4b}}$$

where

(9) $$a=\frac{1}{R_{L}C_{i}}+\frac{1}{R_{L}C_{s}}-\frac{1}{rC_{s}},\;\;b=\frac{c_{3}}{R_{L}{}^{2}C_{s}C_{i}}-\frac{1}{rR_{L}C_{s}C_{i}}$$

*c*_1_, *c*_2_ and *c*_3_ are integration constants. Therefore, the “OFF” photocurrent transient should still exhibit double exponential decay based on Equation (7).

**Figure 4:**
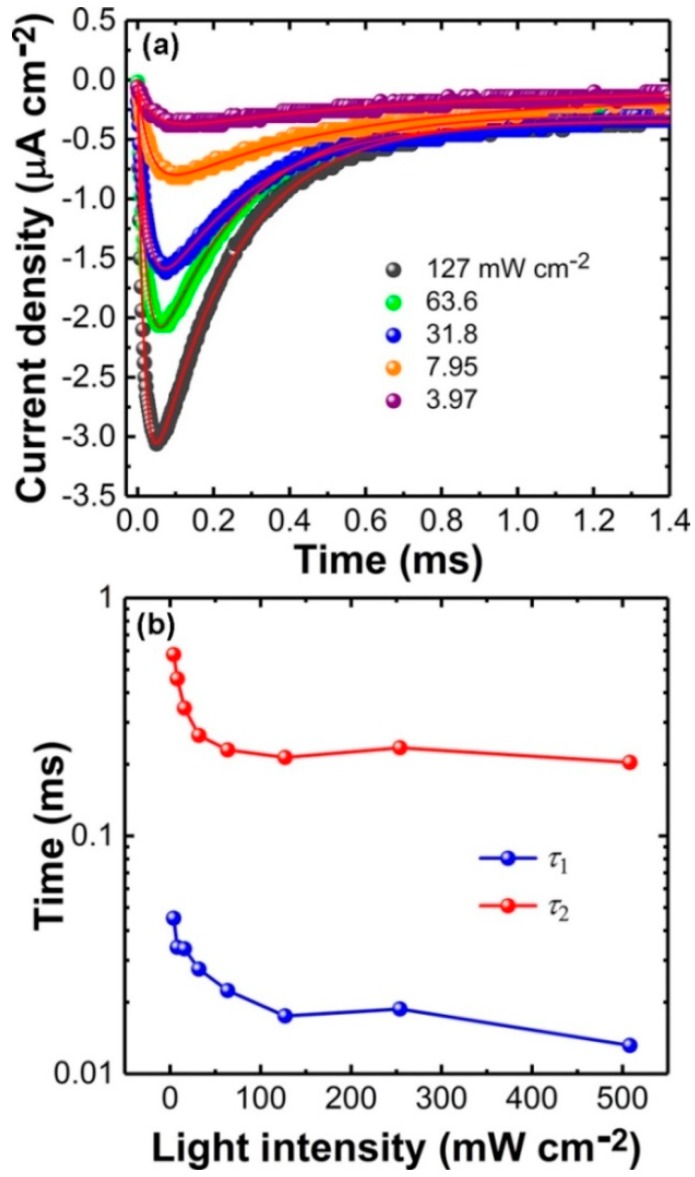
Analyses for the “OFF” photocurrent transients from a ITO/PVDF/ZnPc:C_60_/Al device. (**a**) The “OFF” photocurrent transients after light illumination with different light intensity. Theoretic simulations (red curves) fit the experimental data well. (**b**) Decay time *τ*_1_ (blue points) and *τ*_2_ (red points) at different light intensity can be extracted.

Figure 4a demonstrates the “OFF” transients of the MISM device after laser illumination with different intensity. It was observed that the signals increased with the increase of light intensity and the decay time of the photocurrent transient became faster after a stronger illumination, as did the “ON” transient. To simulate the transient current based on Equation (7), a small current leakage as conduction current was considered in the equation. The simulated curves (red lines) in Figure 4a fit the experimental data well. On the contrary, we failed to simulate the data by using single exponential decay formula, further verifying that the proposed circuit was reasonable and that the two components which are involved in the “OFF” transients. *τ*_1_ (blue points) and *τ*_2_ (red points), could be extracted from the simulation, as is shown in Figure 4b which depicts the relation between *τ*_1,2_ and *I*. Both *τ*_1_ and *τ*_2_ decreased with the increase of light intensity. This was natural, since both *τ*_1_ and *τ*_2_ were related to *r* which was an intensity-dependent value. It was notable that larger intensity (>127 mW cm^−2^) led to saturation with *τ*_1_ and *τ*_2_ around 0.015 and 0.200 ms, respectively. These could be ascribed to the bandwidth limitation of the DPSS laser which can be modulated usually with a frequency about few kHz. In this case, the “OFF” transients with the larger intensity (>127 mW cm^−2^) was determined by the decay process of the laser with a response time of ~10^−4^ s. In addition, all the MISM devices (>5 samples) exhibited similar behavior, i.e., both the “ON” and “OFF” transients consisted of two components and could be fitted by using Equations (1) and (7), when the light intensity was not very strong. We have also applied the equations to other type samples for which the double-layer model is also applicable (e.g., thick 4,4’-bis (1, 2, 3, 5-dithiadiazolyl) (BDTDA) films [26]) and found that the simulated curves could fit well with the experimental data (Supplementary Figure S2).

### 3.4. Photovoltage Reversal

The total photo-generated charges *Q* can be estimated on the basis of the equivalent circuit. Under equilibrium state with continuous illumination, no charging/discharging current could be observed. As both the IL and SL capacitors were charged and the voltages drop caused by the photo-generated charges, will be the same across them, i.e., *Q_s_*/*C_s_* = *Q_i_*/*C_i_*, therefore, the *Q* stored in the two capacitors could be estimated by:

(10) $$Q=Q_{s}+Q_{i}=\left(1+\frac{d_{i}{𝜀}_{s}\;}{d_{s}{𝜀}_{i}}\right)Q_{i}$$

Since *ε_i_* is usually adopted to be 10 and *ε_s_* is about 4, *Q* ≈ 14.3*Q_i_*. For the cases with high light intensity at which saturation of charge extraction can be achieved, *Q_i_* was found to be around 2.0 × 10^−11^ C for the “ON” transients and 1.5 × 10^−11^ C for the “OFF” transients by integrating the transients, respectively. Leakage currents were subtracted for integration. It was notable that all the extracted charges for the “ON” transients were slightly larger than those for the “OFF” transients, indicating that a small part of the photo-generated charges recombined through a small DC photocurrent inside the devices.

**Figure 5:**
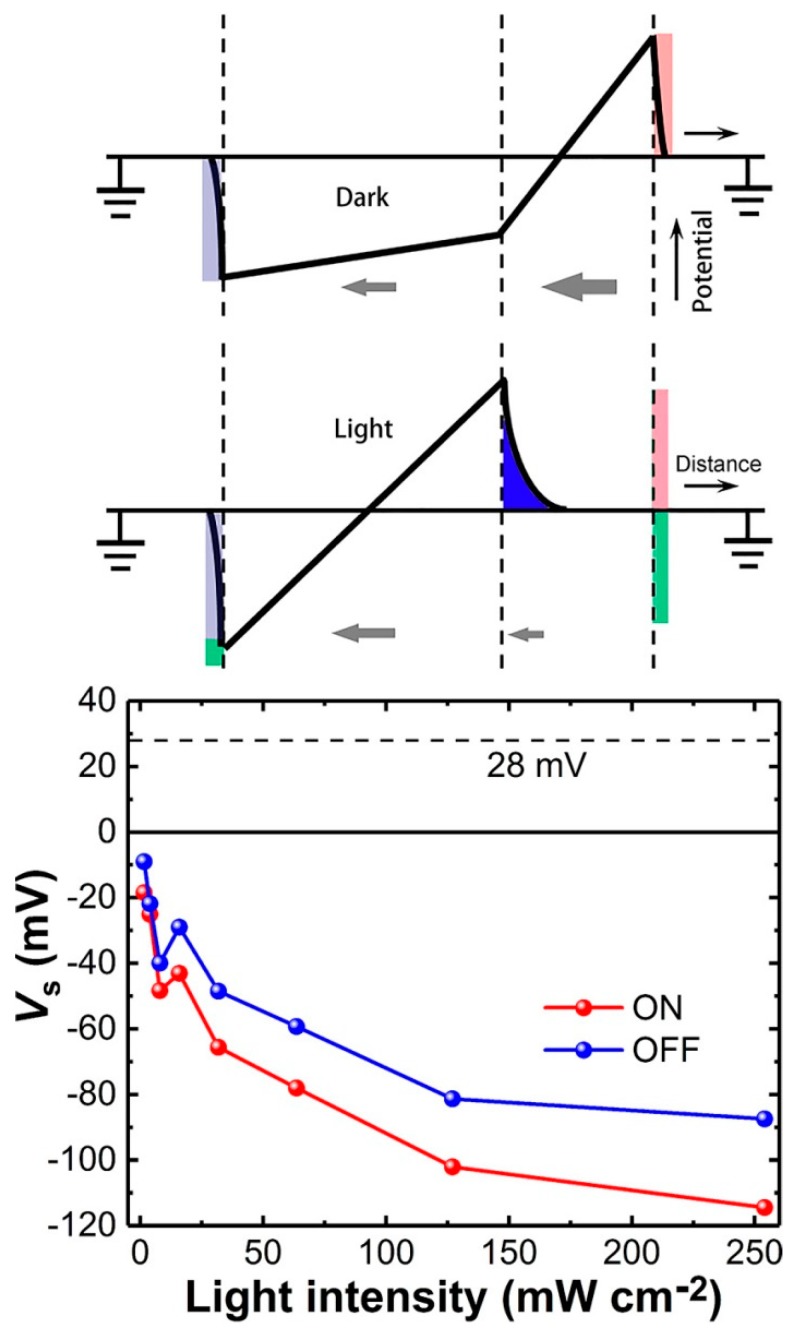
Light intensity dependence of the photoinduced voltages (*V_s_*) drop across the SL capacitor. The inset is a schematic view for the MISM device under dark and light conditions.

Based on Equation (10) and the purely light-induced voltage of the SL capacitor *V_s_* = *Q_s_*/*C_s_*, the *V_s_* at different *I* could be estimated, as shown in Figure 5. For comparison, the original built-in voltage (*E_s_d_s_*, see the black line) caused by the electrodes across the SL, could be calculated to be about 28 mV with an opposite polarity based on their relation to each other, i.e., *V* (0) = *E_s_d_s_* + *E_i_d_i_* and *ε_s_E_s_* = *ε_i_E_i_*, where *E_s_*_,*i*_ is the original built-in electric field in the SL or IL. Here, the polarity of the original built-in field was defined as positive. Considering that there could be plenty of randomly-distributed interfacial dipoles [27] due to the existence of ZnPc/C_60_ interfaces which lead to a larger static permittivity for the blend layer, the *E_s_* would be even smaller and would induce a voltage smaller than 28 mV. The net voltage drop (i.e., photovoltage) across the SL could gradually decrease to zero with an increase in light intensity. It is worth noting that the polarity of the net voltage would change to negative if *I* > 4 mW cm^−2^. The magnitude of the *V_s_* could even exceed the original built-in voltage, suggesting that, under a strong illumination of a 100mW cm^−2^, the photoinduced charges would significantly change the *E_s_*, especially for the organic photovoltaic devices with dielectric/ferroelectric components [20,21,22,23,24].

The insets of Figure 5 clearly demonstrate the underlying physics for the photocurrent transients. Before illumination, the *E_s_* and *E_i_* were uniformly distributed in their respective layers, in the short circuit condition. Original electrons and holes stored in the ITO and Al electrodes are denoted with the gray area and red area, respectively. With strong illumination, the *E_s_* in the bulk region of the SL can even be canceled by the *V_s_* due to the accumulation of photo-generated holes at the IL/SL interface in the form of screening charges (denoted with the blue area). The green area denotes photo-generated electrons distributed in both the electrodes. However, the screening length of organic semiconductors are quite large, usually less than 5–20 nm [28,29], therefore, charge extraction could still occur due to the incomplete screening of the built-in fields of the accumulation layer [15]. Electrons would continue to be extracted from the accumulation layer and would diffuse towards the cathode in the bulk region until equilibrium state has been achieved, in spite of a zero field or even a negative field in the bulk region. For the latter, the opposite fields in the bulk of the SL could also contribute to exciton dissociation and induce an opposite drift photocurrent if they were large enough to overcome the binding energy of charge transfer excitons. Therefore, it was natural that more photo-generated charges could be collected by the cathode with the magnitude of the negative *V_s_* exceeding the original built-in voltage value. Considering that the integration range for the transients to estimate *V_s_* was smaller than the actual range, the actual photo-generated charges and *V_s_* should have been even larger. It should be pointed out that the phenomenon would be universal in organic devices for photoelectric conversion, including photodetectors and phototransistors with large internal differences of permittivity or conductivity discontinuity, in which hetero-structures or bulk-hetero-junctions are always employed. It might also occur in ferroelectric materials due to the existence of domain wall/domain interfaces which can exhibit anomalous photovoltaic effect with a photovoltage exceeding their bandgap [30,31,32].

## 4. Conclusions

In summary, the “ON” and “OFF” photocurrent transients from MISM devices were analyzed theoretically and experimentally. Both the transients were found to involve two different components. Additionally, it was found that they can both be simulated with derived double exponential decay formulae, which is consistent with the proposed equivalent circuit in which the SL and IL capacitors are connected in parallel. Our results clearly demonstrate that photoinduced space-charges in organic semiconductor can significantly suppress or screen built-in fields in most regions. However, the fields in the accumulation layer or space-charge layer at the dielectric/semiconductor interfaces cannot be completely screened due to a large screening length of organic semiconductors. The fields inside the space-charge layer may still be used for charge separation and extraction. This could be promising for interfacial photoelectric conversion in organic systems, such as the devices with dielectric/ferroelectric components, organic phototransistors with gate dielectrics, and artificial bioelectronic devices with various interfaces, including water/semiconductor interfaces. In addition, our results also give a deeper understanding for the organic devices with dielectric/ferroelectric components.

## Supplementary Materials

The following are available online at http://www.mdpi.com/1996-1944/11/9/1530/s1, Figure S1: Photocurrent-action spectrum, Figure S2: Photocurrent transients from other types of devices.
