# Supplementary material for: Photovoltage Reversal in Organic Optoelectronic Devices with Insulator-Semiconductor Interfaces
Source: Materials (Basel). 2018 Aug 25;11(9):1530. doi: 10.3390/ma11091530 (PMC6163970; doi:10.3390/ma11091530)
Supplement: Supplementary file 1 [file materials-11-01530-s001.pdf]

Supplementary Materials

Photovoltage Reversal in Organic Optoelectronic Devices with Insulator-Semiconductor Interfaces

Laigui Hu [1,*], Wei Jin [1], Rui Feng [1], Muhammad Zaheer [1], Qingmiao Nie [2,*], Guoping Chen [1], Zhi-Jun Qiu [1], Chunxiao Cong [1] and Ran Liu [1]

[1] School of Information Science and Technology, Fudan University, Shanghai 200433, China; 17210720055@fudan.edu.cn (W.J.); 16210720056@fudan.edu.cn (R.F.); 17110720056@fudan.edu.cn (M.Z.); gpchenapple@fudan.edu.cn (G.C.); zjqiu@fudan.edu.cn (Z.J.Q.); cxcong@fudan.edu.cn (C.C.); rliu@fudan.edu.cn (R.L.)

[2] Department of Applied Physics, Zhejiang University of Technology, Hangzhou 310023, China;

* Correspondence: laiguihu@fudan.edu.cn (L.H); nieqingmiao@zjut.edu.cn (Q.N.)

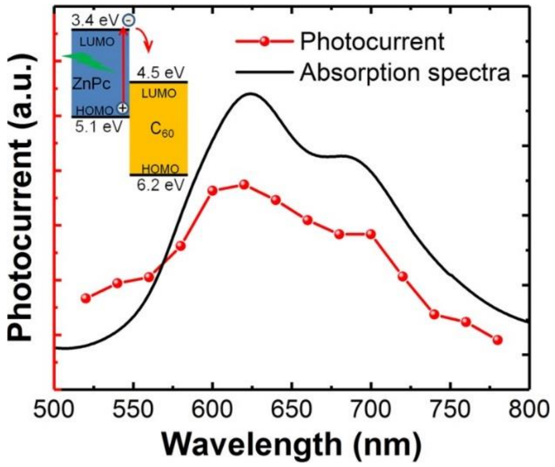

Figure S1: Photocurrent-action spectrum of the MISM device (red line). It matches well with the absorption spectrum of the ZnPc:C_{60} blend films (black line), suggesting that charge separation process mainly occurs in the blend films. Monochromated light from a halogen lamp was utilized as the light source. Photocurrent was collected by a Keithley source meter. The inset exhibits the charge separation process in the blend film upon an illumination of green light.

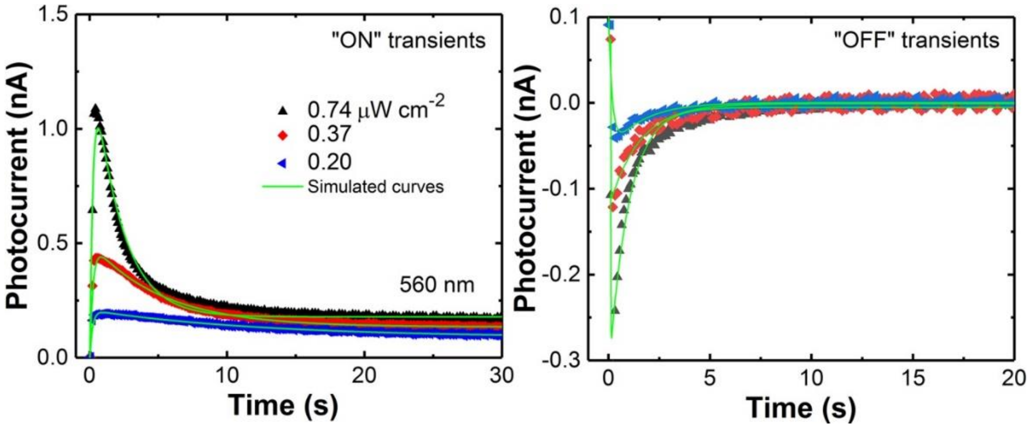

Figure S2: Photocurrent transients from a thick BDTDA film (the data was from Reference [26]) which can be regarded as a double-layer system. Both the "ON" and "OFF" transients can be simulated based on Equations (1) and (7).
